# *Trans*-acting genetic variants causing multilocus imprinting disturbance (MLID): common mechanisms and consequences

**DOI:** 10.1186/s13148-022-01259-x

**Published:** 2022-03-16

**Authors:** Thomas Eggermann, Elzem Yapici, Jet Bliek, Arrate Pereda, Matthias Begemann, Silvia Russo, Pierpaola Tannorella, Luciano Calzari, Guiomar Perez de Nanclares, Paola Lombardi, I. Karen Temple, Deborah Mackay, Andrea Riccio, Masayo Kagami, Tsutomu Ogata, Pablo Lapunzina, David Monk, Eamonn R. Maher, Zeynep Tümer

**Affiliations:** 1 Institute of Human Genetics, Medical Faculty, RWTH Aachen University, Pauwelsstr. 30, 52074 Aachen, Germany; 2 Department of Human Genetics, Laboratory for Genome Diagnostics, Amsterdam UMC, Amsterdam, Netherlands; 3 Molecular (Epi)Genetics Laboratory, Bioaraba Health Research Institute, Hospital Universitario Araba-Txagorritxu, Vitoria-Gasteiz, Alava Spain; 4 Research Laboratory of Medical Cytogenetics and Molecular Genetics, Istituto Auxologico Italiano, IRCCS, Milan, Italy; 5 Department of Medical Genetics, University of Cambridge and Cambridge University Hospitals NHS Foundation Trust, Cambridge, CB2 0QQ UK; 6 Cambridge University Hospitals NHS Foundation Trust, Cambridge, CB2 0QQ UK; 7 Wessex Clinical Genetics Service, University Hospital Southampton, Southampton, UK; 8 Wessex Clinical Genetics Service, University Hospital Southampton NHS Foundation Trust, Southampton, UK; 9 Department of Environmental, Biological and Pharmaceutical Sciences and Technologies, University of Campania ‘Luigi Vanvitelli’, Caserta, Italy; 10 Institute of Genetics and Biophysics ‘Adriano Buzzati–Traverso’ CNR, Naples, Italy; 11 Department of Molecular Endocrinology, National Research Institute for Child Health and Development, Ohkura, Setagayaku, Tokyo, Japan; 12 Department of Pediatrics, Hamamatsu Medical Center, Hamamatsu, Japan; 13 Department of Biochemistry, Hamamatsu University School of Medicine, Hamamatsu, Japan; 14 CIBERER-ISCIII and INGEMM, Institute of Medical and Molecular Genetics, Hospital Universitario La Paz, Madrid, Spain; 15 ERN-Ithaca, European Reference Networks, Madrid, Spain; 16 School of Biological Sciences, University of East Anglia, Norwich, UK; 17 Department of Clinical Genetics, Kennedy Center, Copenhagen University Hospital, Rigshospitalet, Copenhagen, Denmark; 18 Department of Clinical Medicine, Faculty of Health and Medical Sciences, University of Copenhagen, Copenhagen, Denmark

**Keywords:** Imprinting disorders, Differentially methylated regions, Multi locus imprinting disturbance, Uniparental disomy, Growth disturbances, Epimutations, Loss of methylation, Gain of methylation, Beckwith–Wiedemann syndrome spectrum, Silver–Russell syndrome spectrum, Transient neonatal diabetes mellitus

## Abstract

**Background:**

Imprinting disorders are a group of congenital diseases which are characterized by molecular alterations affecting differentially methylated regions (DMRs). To date, at least twelve imprinting disorders have been defined with overlapping but variable clinical features including growth and metabolic disturbances, cognitive dysfunction, abdominal wall defects and asymmetry. In general, a single specific DMR is affected in an individual with a given imprinting disorder, but there are a growing number of reports on individuals with so-called multilocus imprinting disturbances (MLID), where aberrant imprinting marks (most commonly loss of methylation) occur at multiple DMRs. However, as the literature is fragmented, we reviewed the molecular and clinical data of 55 previously reported or newly identified MLID families with putative pathogenic variants in maternal effect genes (*NLRP2*, *NLRP5*, *NLRP7*, *KHDC3L*, *OOEP*, *PADI6*) and in other candidate genes (*ZFP57*, *ARID4A*, *ZAR1*, *UHRF1*, *ZNF445*).

**Results:**

In 55 families, a total of 68 different candidate pathogenic variants were identified (7 in *NLRP2*, 16 in *NLRP5*, 7 in *NLRP7*, 17 in *PADI6*, 15 in *ZFP57*, and a single variant in each of the genes *ARID4A*, *ZAR1*, *OOEP*, *UHRF1*, *KHDC3L* and *ZNF445*). Clinical diagnoses of affected offspring included Beckwith–Wiedemann syndrome spectrum, Silver–Russell syndrome spectrum, transient neonatal diabetes mellitus, or they were suspected for an imprinting disorder (undiagnosed). Some families had recurrent pregnancy loss.

**Conclusions:**

Genomic maternal effect and foetal variants causing MLID allow insights into the mechanisms behind the imprinting cycle of life, and the spatial and temporal function of the different factors involved in oocyte maturation and early development. Further basic research together with identification of new MLID families will enable a better understanding of the link between the different reproductive issues such as recurrent miscarriages and preeclampsia in maternal effect variant carriers/families and aneuploidy and the MLID observed in the offsprings. The current knowledge can already be employed in reproductive and genetic counselling in specific situations.

**Supplementary Information:**

The online version contains supplementary material available at 10.1186/s13148-022-01259-x.

## Background

Imprinting disorders are a group of congenital diseases, which are characterized by molecular alterations affecting differentially methylated regions (DMRs) and/or disrupted regulation of genes that are expressed in a parent-of-origin specific manner, namely the imprinted genes. To date, 12 imprinting disorders with OMIM numbers have been defined (Table 1), and although clinically heterogeneous, some imprinting disorders such as Silver–Russell syndrome (SRS), Beckwith–Wiedemann syndrome (BWS), Temple syndrome (TS14), Kagami–Ogata syndrome (KOS14) and transient neonatal diabetes mellitus (TNMD) have overlapping features such as growth and metabolic disturbances, cognitive dysfunction, abdominal wall defects and asymmetry (for review: [1, 2]). Some of these disorders (BWS and KOS14) are also associated with an increased risk for (embryonal) tumours. Though each imprinting disorder has characteristic disturbances at specific DMRs (so-called imprinting centres, IC), an increasing number of studies report molecular overlaps between these disorders [2]. This overlap indicates a close link in regulation [3] and function of imprinted gene clusters (e.g. [2]).

**Table 1:** Overview on the 12 known imprinting disorders and the ratio of MLID in specific molecular subgroups

Imprinting disorder (abbreviation)	OMIM	Chromosome	Primary epimutation (frequency)^a^	MLID frequency observed for the respective epimutation^b^
Transient neonatal diabetes mellitus (TNDM)	601410	Chr 6q24	*PLAGL1*:alt-TSS-DMR LOM (30%)	30%
Silver–Russell syndrome (SRS)	180860	Chr 11p15	*H19/IGF2*:IG-DMR LOM (30–60%)	7–10%
Birk–Barel syndrome (BIBARS)	612292	Chr 8q24.3	Epimutation not yet reported	–
Beckwith–Wiedemann syndrome (BWS)	130650	Chr 11p15	*KCNQ1OT1*:TSS-DMR LOM (50%)	25%
			*H19/IGF2*:IG-DMR GOM (5–10%)	–
Kagami–Ogata syndrome (KOS14)	608149	Chr 14q32	*MEG3/DLK1*:IG-DMR GOM (15%)	–
Temple syndrome (TS14)	616222	Chr 14q32	*MEG3/DLK1*:IG-DMR LOM (18.8%)	Unclear^c^
Prader–Willi syndrome (PWS)	176270	Chr 15q11–q13	*SNURF*:TSS-DMR GOM (1%)	1 case
Angelman syndrome (AS)	105830	Chr 15q11–q13	*SNURF*:TSS-DMR LOM (2–3%)	–
Central precocious puberty 2 (CPPB2)	615346	Chr 15q11.2	Epimutation not yet reported	–
Schaaf–Yang syndrome (SYS)	615547	Chr 15q11.2	Epimutation not yet reported	–
Pseudohypoparathyroidism 1B (PHP1B)	603233	Chr 20q13	Maternal *GNAS* DMRs LOM with paternal *GNAS* DMR GOM (42.5%)	12.5%
Mulchandani–Bhoj–Conlin syndrome (MBCS)	617352	Chr 20	Epimutation not yet reported	–

^a^Frequency of the epimutation among other genetic/epigenetic changes causative for a given disorder. LOM, loss of methylation; GOM, gain of methylation

^b^Reviewed by [4]

^c^Some TS14 patients have been reported with aberrant methylation at imprinted loci, but in these patients clinically relevant CpGs were not affected with the exception of those in 14q32 [18, 38]

Imprinting disorders may be caused by a variety of genetic alterations such as pathogenic variants in imprinted genes, copy number abnormalities and uniparental disomy. For some imprinting disorders, the primary molecular mechanism is epimutations (imprinting defects, namely gain or loss of methylation (GOM or LOM) at an imprinting center [1]. Epimutations have been frequently observed as primary events without presence of obviously detectable genetic alterations. However, in some cases they were secondary to genetic alterations such as copy number variations (CNV) or single nucleotide variations (SNVs) within the DMR or secondary to inactivating variants in trans-acting factors with a key role in the establishment or maintenance of methylation status of an IC. Epimutations have been identified in eight of the 12 imprinting disorders and usually affect a single specific locus for a given condition (Table 1). However, there are a growing number of reports of individuals with so-called multilocus imprinting disturbances (MLIDs), whereby aberrant imprinting marks (most commonly LOM) occur at multiple DMRs (reviewed in Additional file 1: Table S1). MLID is frequently detected in individuals with TNDM, BWS spectrum (BWSp) and SRS spectrum (SRSp) [4], but it appears to be rare in the other imprinting disorders. Notably, with the exception of TNDM [5], the presence of MLID can result in discordance between the epigenotype and clinical phenotype. For example, one of the primary epimutations associated with BWSp is GOM at *H19/IGF2*:IG-DMR (IC1) on the maternal allele. However, with the presence of MLID an individual with BWSp symptoms might have LOM at this locus, where LOM of IC1 is normally associated with SRSp. A plausible explanation is that methylation patterns can differ in different tissues of the same individual as observed for SRSp, and this mosaic distribution might explain the divergent clinical features of individuals with the same blood methylation patterns [6, 7].

**Fig. 1:**
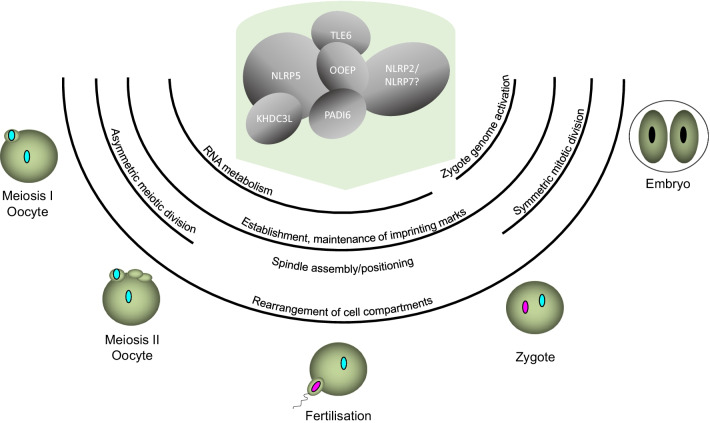
Factors and function of the SCMC (from [43])

As mentioned above, rare cases of CNVs or SNVs may affect genomic regions or transcription of genes close to the DMRs leading to epimutations. These *cis*-acting regions or gene transcripts are involved in the establishment or maintenance of the imprinting marks, as recently shown for the CTCF binding sites of the imprinting center *H19/IGF2*:IG-DMR (IC1) or the alterations of *KCNQ1* transcript regulating the *KCNQ1OT1*:TSS-DMR (IC2) [8, 9]. In fact, these *cis*-acting elements are required for the proper imprinting marks of specific loci, but at least some reports of MLID indicate that also higher-order mechanisms orchestrate the coordinated episignature of a network of imprinted genes [10]. *Trans*-acting causes of secondary epimutations can currently be identified in approximately 30% of MLID families (T. Eggermann, personal communication) and include loss-of-function variants in *NLRP2*, *NLRP5*, *NLRP7*, *PADI6*, or rarely *KHDC3L* (so-called maternal effect genes) in the asymptomatic mothers of the offspring with MLID (for review: [3]). The proteins encoded by these genes are localized to the subcortical maternal complex (SCMC) which is required for the proper oocyte maturation and early embryonic development (Fig. 1). Maternal effect variants of these genes have been proposed to disrupt the function of SCMC leading to aberrant methylation signatures which can also, in addition to congenital imprinting disorders, be associated with biparental hydatidiform moles (BiHM) and pregnancy loss (for review: [11]). Another *trans*-acting cause of MLID associated with TNMD phenotype is biallelic variants—identified in affected individuals in contrast to maternal effect gene variants—of *ZFP57*, protein product which is involved in protection of methylation in early development (for review: [12]). Non-genetic factors have also been implicated in susceptibility to altered imprinting signatures, including assisted reproductive technologies (ART), monozygotic twinning, parental nutritional and metabolic status and teratogenic substances [13, 14].

In this study, we attempt to delineate the genetic architecture and clinical expressivity of MLID in human imprinting disorders by compiling published and new *trans-*acting genetic causes of epimutations.

## Overview of genes associated with MLID

We gathered molecular and clinical data of 55 families where at least one individual had MLID. Among these families, 21 mothers had biallelic and 15 mothers had heterozygous putative pathogenic variants of *NLRP2*, *NLRP5*, *NLRP7*, *PADI6*, *KHDC3L* or *OOEP.* Biallelic *ZFP57* variants were found in 15 families, and four families had variants in *trans*-acting MLID susceptibility genes (*ARID4A*, *ZAR1*, *UHRF1*, *ZNF445*) (Table 2, Additional file 1: Table S1). Furthermore, we included four unpublished cases (Families 42, 53, 54, 55).

**Table 2:** Summary of genomic variants in the maternal effect and foetal genes associated with MLID

Gene/transcript	Family	Variant (c.DNA)	Genomic position					Protein	dbSNP	GnomAD v2.1.1		CADD v1.6
			Chr	Start hg38	Stop hg38	wt	alt			Allele frequency total	Number of homozygotes	
*NLRP2* NM_017852.4	4	c.314C>T	19	54,974,533	54,974,533	C	T	p.(Pro105Leu)	rs201724086	0.00002785	0	3.863
	1, 6	c.1479_1480del	19	54,983,177	54,983,178		del	p.(Arg493Serfs*32)	rs758760659	0.00007564	0	
	48	c.1870C>T	19	54,983,568	54,983,568	C	T	p.(Gln624*)	No			35
	5	c.1885T>C	19	54,983,583	54,983,583	T	C	p.(Ser629Pro)	rs147213467	0.001019	0	20.7
	2	c.2237del	19	54,986,186	54,986,186		del	p.(Asn746Thrfs*4)	rs1190657804	0.000003977	0	
	5	c.2401G>A	19	54,990,056	54,990,056	G	A	p.(Ala801Thr)	rs117066658	0.009561	18	14.6
	3	c.2860_2861del	19	54,994,420	54,994,421		del	p.(Cys954Glnfs*18)	No			
*NLRP5* NM_153447.4	12	c.68T>A	19	56,003,721	56,003,721	T	A	p.(Val23Asp)	rs753824534	0.000004304	0	17.29
	9	c.155T>C	19	56,003,808	56,003,808	T	C	p.(Met52Thr)	rs752189640	0.000008023	0	6.797
	9	c.226G>C	19	56,003,879	56,003,879	G	C	p.(Glu76Gln)	rs758399773	0.000008023	0	23.1
	54	c.842C>T	19	56,027,075	56,027,075	C	T	p.(Thr281Met)	rs45627733	0.001336	0	21.7
	14	c.1057C>T	19	56,027,290	56,027,290	C	T	p.(Arg353*)	No			34
	51	c.1111C>T	19	56,027,344	56,027,344	C	T	p.(Leu371Phe)	rs191432085	0.0003220	0	0.909
	10	c.1156_1158dup	19	56,538,757	56,538,759		dup	p.(Pro386dup)	rs748872279	0.000004031	0	
	53	c.1588C>T	19	56,027,821	56,027,821	C	T	p.(Arg530Cys)	rs200705062	0.0002531	0	16.3
	14	c.1597C>T	19	56,027,830	56,027,830	C	T	p.(Arg533Cys)	rs754695863	0.00001782	0	22
	7	c.1664G>T	19	56,027,897	56,027,897	G	T	p.(Gly555Val)	No			22.4
	11	c.1699A>G	19	56,027,932	56,027,932	A	G	p.(Met567Val)	rs748718334	0.00004419	0	5.29
	53	c.2090_2091del	19	56,028,323	56,028,324		del	p.(Lys697Argfs*18)	rs771412598	0.0002031	0	
	7	c.2320T>C	19	56,032,654	56,032,654	T	C	p.(Cys774Arg)	rs370837790	0.000004016	0	23.8
	8, 54	c.2353C>T	19	56,032,687	56,032,687	C	T	p.(Gln785*)	rs200446614	0.00008432	0	36
	8	c.2840T>C	19	56,040,975	56,040,975	T	C	p.(Leu947Pro)	rs202181446	0.0002566	0	23.9
	13	c.3259G>A	19	56,053,768	56,053,768	G	A	p.(Glu1087Lys)	rs762535392	0.000008029	0	6.696
*NLRP7* NM_001127255	19	c.574A>C	19	54,940,245	54,940,245	T	G	p.(Met192Leu)	rs104895529	0.001856	4	4.191
	17	c.749T>G	19	54,940,070	54,940,070	A	C	p.(Phe250Cys)	rs78096121	0.0004525	0	23.5
	17	c.1104T>G	19	54,939,715	54,939,715	A	C	p.(Ile368Met)	rs1654636	0.0004910	0	0.162
	19	c.2010_2011del	19	54,938,162	54,938,163		del	p.(Phe671Glnfs*18)	rs1467166317	(0.000006576)^a^	0	
	15, 18	c.2156C>T	19	54,936,405	54,936,405	G	A	p.(Ala719Val)	rs104895526	0.001050	1	18.38
	16	c.2161C>T	19	54,936,400	54,936,400	G	A	p.(Arg721Trp)	rs104895525	0.00005967	0	14.24
	16	c.2573T>C	19	54,933,638	54,933,638	A	G	p.(Ile858Thr)	rs776102152	0.00007070	0	18.16
*PADI6* NM_207421.3	24	c.433A>G	1	17,379,985	17,379,985	A	G	p.(Lys145Glu)	rs1413565869	(0.000006571)^a^	0	23.9
	21	c.902G>A	1	17,388,820	17,388,820	G	A	p.(Arg301Gln)	rs755969432	0.00002010	0	25.2
	23	c.1046A>G	1	17,392,197	17,392,197	A	G	p.(Asp349Gly)	No			23.9
	25	c.1067G>A	1	17,392,218	17,392,218	G	A	p.(Trp356*)	No			55
	20	c.1114A>G	1	17,394,014	17,394,014	A	G	p.(Thr372Ala)	rs374615037	(0.000006571)^a^	0	22.8
	22	c.1124T>C	1	17,394,024	17,394,024	T	C	p.(Leu375Ser)	rs1470278066	0.000004012	0	23.5
	21	c.1298C>T	1	17,394,415	17,394,415	C	T	p.(Pro433Leu)	rs759006424	0.00004427	0	24.7
	26	c.1429A>G	1	17,395,042	17,395,042	A	G	p.(Met477Val)	rs761556429	0.000004008	0	11.96
	51	c.1456T>C	1	17,395,069	17,395,069	T	C	p.(Cys486Arg)	No			23.4
	22, 49	c.1639G>A	1	17,397,091	17,397,091	G	A	p.(Asp547Asn)	rs150981529	0.0005529	0	0.524
	49	c.1663dup	1	17,397,115	17,397,115		dup	p.(Leu555Profs*6)	rs766500048	0.000008031	0	
	52	c.1709G>A	1	17,398,705	17,398,705	G	A	p.(Arg570His)	rs372730186	0.00001455	0	23.4
	51	c.1874dup	1	17,401,227	17,401,227		dup	p.(Asn626Glufs*38)	rs745431993	0.00001204	0	
	25	c.1894C>G	1	17,401,247	17,401,247	C	G	p.(Pro632Ala)	rs755260464	0.000004012	0	25
	27	c.2006del	1	17,401,359	17,401,359		del	p.(Thr669Lysfs*86)	No			
	20, 55	c.2069G>A	1	17,401,422	17,401,422	G	A	p.(Trp690*)	No			50
	26	c.2080C>T	1	17,401,433	17,401,433	C	T	p.(Pro694Ser)	rs1368496637	0.000008050	0	23.4
*ZFP57* NM_001109809.1	33	c.317_318del	6	29,675,420	29,675,421		del	p.(Glu106Valfs*28)	rs606231121	(0.000006573)^a^	0	
	31	c.372del	6	29,673,739	29,673,739		del	p.(Arg125Glufs*7)	rs1344415728	0.000004143	0	33
	29, 30	c.373C>T	6	29,673,738	29,673,738	G	A	p.(Arg125*)	No			0.014
	39, 40	c.458del	6	29,673,653	29,673,653		del	p.(Leu153Hisfs*49)	rs1027550840	0.000004119	0	
	28	c.475A>T	6	29,673,636	29,673,636	T	A	p.(Thr159Ser)	rs1334830817	0.001553	2	0.04
	41	c.742C>T	6	29,673,369	29,673,369	G	A	p.(Arg248Cys)	rs1488922640	0.00001635	0	26.1
	36, 38	c.743G>A	6	29,673,368	29,673,368	C	T	p.(Arg248His)	rs77625743	0.00001449	0	24.1
	42	c.748C>T	6	29,673,363	29,673,363	G	A	p.(Arg250Cys)	rs750705477	0.0025	0	23.5
	32	c.783C>A	6	29,673,328	29,673,328	G	T	p.(Cys261*)	rs61730328			36
	40	c.820C>T	6	29,673,291	29,673,291	G	A	p.(Leu274Phe)	No			19.36
	37	c.829C>A	6	29,673,282	29,673,282	G	T	p.(His277Asn)	rs78378398			25
	38	c.839_846del	6	29,673,265	29,673,272		del	p.(Ile280Lysfs*21)	No			
	28	c.1033G>C	6	29,673,078	29,673,078	C	G	p.(Ala345Pro)	rs200537697	0.001547	2	2.277
	35	c.1372C>G	6	29,672,739	29,672,739	G	C	p.(His458Asp)	rs79020217			24.9
	34	c.1383del	6	29,672,728	29,672,728		del	p.(Tyr462Ilefs*16)	rs606231122			
*ARID4A* NM_002892.3	43	c.1181A>G	5	131,238,707	131,238,707	G	A	p.(Tyr394Cys)	rs575489323	0.00001843	0	3.43
*OOEP* NM_001080507.2	45	c.109C>T	6	73,369,684	73,369,684	G	A	p.(Arg37Trp)	rs189355507	0.00001204	0	25.7
*ZAR1* NM_175619.2	47	c.130G>T	4	48,490,421	48,490,421	G	T	p.(Gly44Cys)	No			20.3
*UHRF1* NM_013282.4	46	c.514G>A	19	4,930,794	4,939,794	G	A	p.(Val172Met)	No			9.791
*KHDC3L* NM_001017361.2	44	c.1A>G	6	73,362,730	73,362,730	A	G	p.?	rs606231235	(0.000006570)^a^	0	22.4
*ZNF445* NM_181489.6	50	c.2803C>T	3	44,446,868	44,446,868	G	A	p.(Gln935*)	No			33

Chr, chromosome; wt, wildtype allele; alt, altered allele

^a^The frequencies according to were gathered from gnomAD version (v3.1.1)

### MLID associated with variants in maternal-effect genes

#### NLRP2

Seven different genomic variants in *NLRP2* were described in seven mothers (F1–6, F48). Three mothers (F1, 6, 48) were homozygous for truncating variants, and two mothers (F1, F6) had the same variant and gave birth to four children with BWSp. Four individuals were heterozygous for truncating (F2, F3) or missense (F4, F5) variants; and three children (F2, F4, F5) were suspected to have SRSp or TNDM, and one child (F3) had growth retardation, microcephaly and 46,XXY karyotype [15]. One homozygous (F1) and one heterozygous (F4) mothers had miscarriages, and further pregnancy complications comprised polyhydramnios, raised ß-HCG levels and a probable HDM in a homozygous mother (F6) (Table 3). One proband (F2) with SRSp was born after ART (intracytoplasmic sperm injection (ICSI)) [15].

**Table 3:** Information on reproductive and history in MLID families with variants in SCMC genes. It should be noted that reproductive and family history was not available for all families. (a) In these families, the mothers were either homozygous or compound heterozygous for maternal effect variants. (b) In the mothers in these families, only one variant could be detected. (only families for which information was provided are listed; gw gestational week; *the variants in these families have already been reported to be associated with BiHDM. aTannorella P, ESHG 2021 meeting, P20.020C)

Gene	Family ID*	Phenotype of the offspring	Zygosity in mother	Abortions/miscarriages	HDM	ART	Preeclampsia	Aneuploidy	Family history of miscarriages	Reference
(a)										
*NLRP2*	1	BWSp, BWSp	Homozygous	3 (gw8, 24, 36)						[15]
	6	BWSp, BWSp	Homozygous		Yes					[23]
	48	BWSp	Homozygous							^a^
*NLRP5*	7	SRSp, BWSp	Compound heterozygous	6						[20]
	8	BWS, unspecific	Compound heterozygous	4			Yes			[20]
	9	BWSp	Compound heterozygous							[20]
	11	Unspecific	Homozygous							[20]
	14	BWSp, healthy	Compound heterozygous	4 (gw12, 23, 23, 29)						[21]
	53	BWSp	Compound heterozygous							Unpublished
	54	BWSp	Compound heterozygous							Unpublished
*NLRP7*	16*	BWSp	Compound heterozygous	2 (gw4, 4)	Yes				Yes	[15]
	17*	BWSp	Compound heterozygous		Yes					[15]
*PADI6*	20	BWSp, BWSp	Compound heterozygous	3 (gw33, gw11, gw?)				69,XXY		[39]
	21	SRSp	Compound heterozygous						Yes	[15]
	22	BWSp	Compound heterozygous							[15]
	25	BWSp, BWSp	Compound heterozygous							[16]
	26	BWSp	Compound heterozygous	1 (gw20)						[16]
	49	BWSp	Compound heterozygous		8					^a^
*PADI6*, *NLRP5*	51	SRSp	Compound heterozygous, heterozygous							[34]
(b)										
*NLRP2*	2	SRSp	Heterozygous			ICSI				[15]
	3	Growth retardation	Heterozygous					47,XXY	Yes	[15]
	4	TNDM	Heterozygous	2						[15]
	5	SRSp	Heterozygous	1						[15]
*NLRP5*	10	SRSp, healthy	Heterozygous							[20]
	12	SRSp	Heterozygous							[40]
	13	SRSp	Heterozygous							[40]
*NLRP7*	15*	Unspecific	Heterozygous	2	Yes					[41]
	18 *	SRSp	Heterozygous		Yes					[15]
	19	BWSp, unspecific	Cis	1			Yes		Yes	[24]
*PADI6*	23	SRSp	Heterozygous							[15]
	24	SRSp	Heterozygous							[15]
	27	BWSp	Heterozygous							[16]
	52	SRSp	Heterozygous							[42]
	55	BWSp	Heterozygous							Unpublished

Five of the seven variants were reported in gnomAD, but homozygosity was described only for c.2401G>A, p.(Ala801Thr) (18 times). Apart from the variant c.1479_1480del, p.(Arg493Serfs*32) which occurred in two unrelated families (F1, F6) no other variant was recurrent. Three of the alterations were frameshift variants, one was a nonsense variant. Of the three missense variants, one had a CADD Phred score higher than 20.

**Fig. 2:**
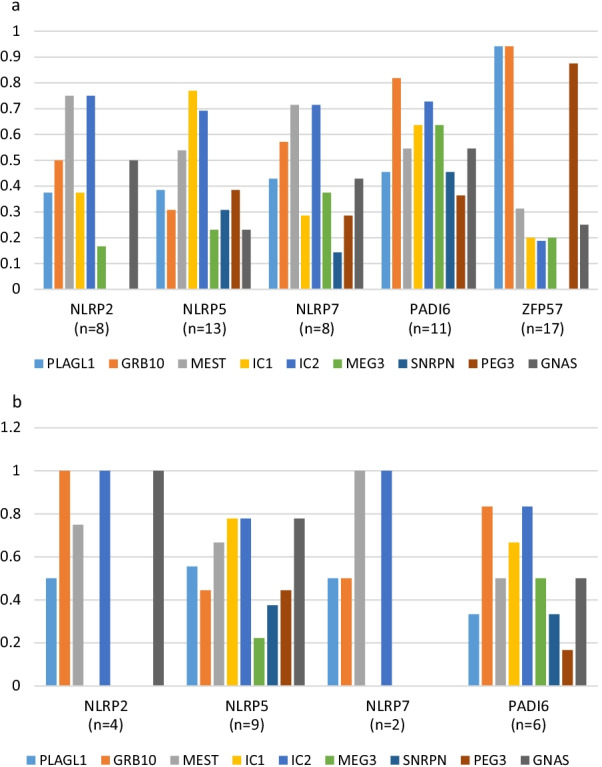
Relative distribution (Y axis) of LOM at the clinically relevant DMRs in correlation to the maternal effect and *ZFP57* genes. (Please note that abbreviated names for the DMRs are listed). **a** Distribution of LOM in the whole cohort. **b** Imprinting signature in those families in which biallelic and homozygous maternal variants have been identified. Only individuals for whom methylation data were available were included. *y*-axis indicates the number of the individuals. PLAGL1, *PLAGL1*:alt-TSS-DMR; GRB10, *GRB10*:alt-TSS-DMR; MEST, *MEST*:alt-TSS-DMR; IC1, *H19/IGF2*:IG-DMR; IC2, *KCNQ1OT1*:TSS-DMR; MEG3, *MEG3/DLK1*:IG-DMR; SNRPN, *SNURF*:TSS-DMR; PEG3, *PEG3*:TSS-DMR; GNAS, *GNAS* DMRs

The majority of MLID individuals from the *NLRP2*-associated families exhibited LOM at *MEST* and IC2 (Fig. 2a). LOM of *GRB10* and *GNAS* was observed in half of the analysed individuals. *PLAGL1* and IC1 were affected in 37.5% of cases. Other loci were not affected or only once.

#### NLRP5

In *NLRP5*, 16 different variants were identified in 11 families. Six mothers were compound heterozygous (F7-F9, F14, F53, F54) and one homozygous (F11). Four mothers were heterozygous (F10, F12, F13, F51). The mother (F51), heterozygous for a missense variant, also had biallelic *PADI6* variants.

Six children were referred with BWSp features (F7–9, 14, 53, 54), five for SRSp features (F7, F10, F12–14) and two children had unspecific phenotypes (F8, F11). Two children of a compound heterozygote (F10) and a heterozygote mother (F14) were described as healthy; they were siblings of MLID individuals with SRSp or BWSp, respectively. Notably, one compound heterozygote mother (F7) gave birth to two children, one with SRSp and the other with BWSp phenotype. Three of the mothers with biallelic variants had miscarriages (F7, F8, F14), but none of the heterozygotes (Table 3). Preeclampsia was reported in one mother (F8).

Fourteen variants were reported in gnomAD in heterozygous form, but none of them were in homozygous form. With the exception of c.2353C>T (p.(Gln785*), all variants occurred only once in the cohort. Four variants were truncating variants (two nonsense and two frameshift), while the remaining twelve were missense variants, six of which had a CADD Phred score higher than 20.

The majority of MLID individuals (69.2%) from the families with *NLRP5* variants presented LOM of IC1, and four of them with SRSp and six with BWSp phenotype. The next most common methylation change was *MEST* LOM (53.8%) and LOM of *PLAGL1* (45.5%). Other loci were affected less frequently (Fig. 2a).

#### NLRP7

Seven different *NLRP7* variants were described in five families (F15–F19). In two families, mothers were compound heterozygous (F16, F17) and they each had a child with BWSp features. One of these children (F16) was ascertained at 19 weeks of gestation with macroglossia and placental mesenchymal dysplasia; and the mother had two further pregnancy losses. The children of the three heterozygous mothers exhibited BWSp (F19), SRSp (F18) or unspecific phenotypes (F15). In family 15, the first child was deceased and the mother also had miscarriages. Notably, two compound heterozygous (F16, F17) and two heterozygous (F15, F18) mothers had HDM (Table 3).

All the variants, except for a single frameshift variant, were missense, and they were reported in gnomAD. Two of the missense variants were reported in homozygous form in gnomAD: c.574A>C, p.(Met192Leu) (four times) and c.2156C>T, p.(Ala719Val) (once). The latter variant was detected twice in the MLID cohort, whereas the others were not recurrent. Only one of the missense variants had a CADD Phred score higher than 20.

The most commonly hypomethylated loci were *MEST* and IC2. LOM of *GRB10* was observed in 57.1%, and LOM of *PLAGL1* and *GNAS* each in 42.8% of individuals. LOM of *MEG3* occurred in 37.5% of the probands. Other loci were affected less frequently (Fig. 2a).

#### PADI6

Seventeen different genomic variants in *PADI6* were identified in 12 mothers. Seven mothers were compound heterozygous for *PADI6* variants, one of them was also heterozygous for an *NLRP5* variant (F51). They gave birth to eight children with BWSp (F20, 22, 25, 26, 49) and one with SRSp (F21). Two of the five heterozygous mothers had children with BWSp (F27, F55) and three with SRSp features (F23, F24, F52). Three of the mothers had miscarriages; two of them were compound heterozygous (F20, F26); and one was heterozygous (F55) (Table 3).

Of the 17 variants, 12 were missense, 10 of which were reported in gnomAD in heterozygous form. Furthermore, two truncation variants were also reported in gnomAD. None of these were reported in homozygous form. With the exception of c.1639G>A, p.(Asp547Asn) and c.2069G>A, p.(Trp690*), all the variants were detected only once. Of the five truncating variants, two were nonsense and frameshift variants. Ten of the missense variants had a CADD Phred score higher than 20.

The locus most frequently affected by LOM was *GRB10*. LOM at IC1, IC2 and *MEG3* was each observed in 70% of the individuals. *MEST* and *GNAS* were altered in 60.0% of the individuals, 50% exhibited LOM at *SNRPN*, 40% at *PLAGL1* and 30% at *PEG3* (Fig. 2a). In one family (F26), GOM of *PLAGL1* was reported [16].

#### KHDC3L

The first and up to date only maternal effect variant in *KHDC3L* associated with MLID has recently been reported by Demond et al. [17] (F44). In this consanguineous family, the mother was homozygous for an SNV affecting the translation initiation codon (c.1A> G) with a CADD Phred score of 22.4. MLID was identified in the preimplantation embryo and the molar tissue.

#### Further maternal effect candidate genes

In addition to the aforementioned factors, *OOEP*, *ARID4*, *ZAR1* and *UHRF2* have been suggested as further putative candidate genes [15, 18]; however, these findings require further confirmation before considering them in the clinical practice.

### Autosomal recessive gene variants associated with MLID

Until now, the only exemplar for this group of conditions is *ZFP57* (see below); however, *ZNF445* is also a strong candidate as an MLID susceptibility gene. In mice, ZNF445 acts with ZFP57 to maintain methylation at most imprinting control regions and Kagami et al. [19] reported a homozygous nonsense *ZNF445* variant in a child with Temple syndrome and MLID (F50).

#### ZFP57

A total of 15 different *ZFP57* variants were ascertained in a total of 16 affected individuals (including two siblings). TNDM was the clinical diagnosis of 15 individuals. The child of family 42 (own unpublished data) was referred for molecular BWS testing. Fourteen patients were homozygous and two were compound heterozygous.

Three variants have been published twice in TNDM/MLID patients. Five variants were frameshift variants, two were nonsense, and eight were missense alterations. Among the latter, seven had a CADD Phred score higher than 20. Eight variants were reported in gnomAD and homozygosity was reported for c.475A>T, p.(Thr159Ser) and c.1033G>C, p.(Ala345Pro), twice for each.

The majority of *ZFP57* associated MLID individuals exhibited LOM at three imprinted loci: *PLAGL1* as the phenotype determining DMR, *GRB10* and *PEG3* (Fig. 2a). Further loci were found to be affected as well, but not all of them have been analysed in the different studies, and hence their frequency is yet unknown. Notably, the proband of the above-mentioned family (F42) who was referred with BWSp features exhibited the characteristic LOM signature linked to *ZFP57* variants.

### Genotype-epigenotype correlation

A correlation between the mutated gene and a specific epigenotype in the foetus/offspring is rather clear for *ZFP57* (Fig. 2a): More than 90% of the individuals reported so far show LOM of *PLAGL1* and *GRB10*, and LOM of *PEG3* is also frequently observed.

For the SCMC-related genes, similar correlations are less obvious. In fact, the majority of MLID families carrying *NLRP2*, *NLRP5*, *NLRP7* and *PADI6* variants have been ascertained with clinical features of BWSp or SRSp. Accordingly, LOM of IC1 and IC2 is frequently observed in the cohort.

By comparison of the mean numbers of aberrant imprinting marks per gene, it appears as if individuals with MLID due to *PADI6* variants exhibit a larger number of epimutations than those associated with the variants in *NLRP* genes (Fig. 2a). However, this should be taken with caution due to the limited number of cases and different methods employed for methylation analysis. When taking a single imprinted locus into consideration *MEST* and *GNAS* are the DMRs which are most frequently hypomethylated, independently of the gene causing MLID.

A comparison between the families with biallelic maternal effect variants (*n* = 20 families) and monoallelic variants (*n* = 19 families) did not reveal clear differences in the epimutation signature (Fig. 2b) but the comparison was limited by genetic heterogeneity and the small number of cases.

### Genotype–phenotype correlation

**Fig. 3:**
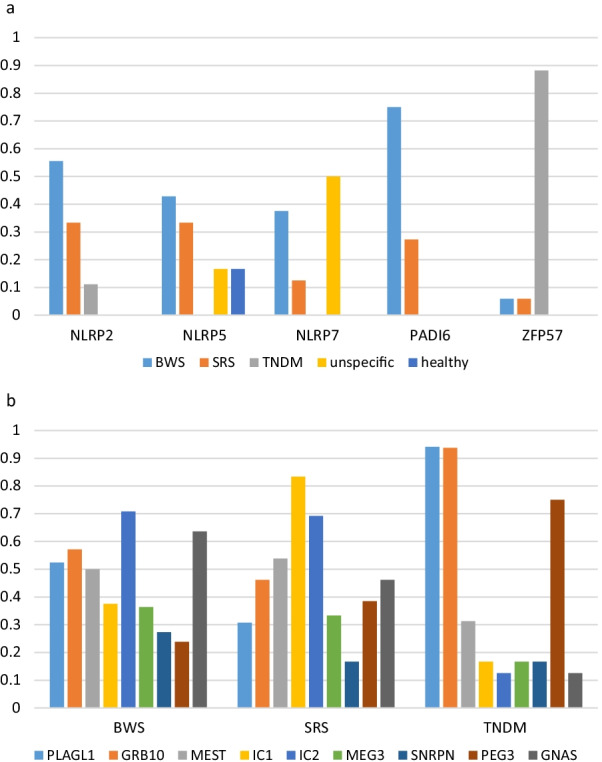
(Epi)genotype–phenotype correlation of the maternal-effect and *ZFP57* genes. **a** Correlation between genes and clinical pictures. **b** correlation between affected DMRs and clinical pictures. (please note that abbreviated names for the DMRs are listed). Only individuals for whom methylation data were available were included. *y*-axis indicates the number of the individuals. PLAGL1, *PLAGL1*:alt-TSS-DMR; GRB10, *GRB10*:alt-TSS-DMR; MEST, *MEST*:alt-TSS-DMR; IC1, *H19/IGF2*:IG-DMR; IC2, *KCNQ1OT1*:TSS-DMR; MEG3, *MEG3/DLK1*:IG-DMR; SNRPN, *SNURF*:TSS-DMR; PEG3, *PEG3*:TSS-DMR; GNAS, *GNAS* DMRs

The confirmed MLID-associated genes were associated with a range of clinical phenotypes including BWSp, SRSp, TNDM and non-specific phenotypes (Fig. 3a, Additional file 1: Table S1). However, *ZFP57* variants were mainly identified in individuals with TNDM, whereas variants in maternal effect genes were associated with SRSp or BWSp features. Children of mothers with *NLRP7* variants frequently present with non-specific phenotypes and in two *NLRP5* families probands were asymptomatic despite MLID (F10, F14) [20, 21].

Families with reproductive issues such as BiHDM and pregnancy loss were outside the scope of this review, but in the families ascertained because of a child with MLID, the typical reproductive problems associated with pathogenic maternal effect variants were also present (Table 3a, b). Thirteen mothers from MLID families had miscarriages that were occasionally recurrent, and among them seven had biallelic and six heterozygous variants in maternal effect genes. In two of these families (F16, F19), further relatives were affected by recurrent miscarriages. In two other families (F31, F21), close relatives, but not the carrier mothers, had miscarriages. HDM was documented in six families: four mothers had biallelic and two heterozygous variants. In five of these families, variants in *NLRP7* and *KHDC3L*, the two genes which are associated with BiHDM, were detected. Nearly all *NLRP7* variants detected in mothers experiencing HDM have also been described in cohorts of (recurrent) BiHDM families (p.(Phe250Cys) (F17), p.(Ala719Val) (F18), p.(Arg721Trp) (F16), p.(Ile858Thr) (F16) (see https://infevers.umai-montpellier.fr/web/). Preeclampsia was observed in two families (F8, F19). One proband was conceived by ART (F2). In two families with variants in maternal effect genes, two offsprings had aneuploidy (F3, F20). When families with biallelic or heterozygous variants in maternal affect genes were compared (Table 3a, b), there were no clear differences in the reproductive histories. However, this should be taken cautiously due to the small numbers of individuals/families.

### Epigenotype-phenotype correlation

**Fig. 4:**
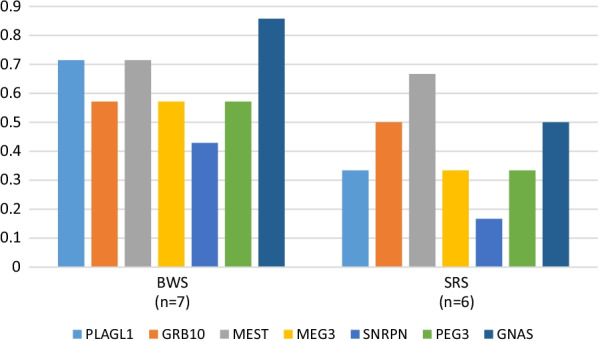
Overview on the DMRs affected in MLID individuals in which both ICs in 11p15.5 are affected. PLAGL1, *PLAGL1*:alt-TSS-DMR; GRB10, *GRB10*:alt-TSS-DMR; MEST, *MEST*:alt-TSS-DMR; IC1, *H19/IGF2*:IG-DMR; IC2, *KCNQ1OT1*:TSS-DMR; MEG3, *MEG3/DLK1*:IG-DMR; SNRPN, *SNURF*:TSS-DMR; PEG3, *PEG3*:TSS-DMR; GNAS, *GNAS* DMRs

Among the individuals referred with clinical suspicion of BWS (*n* = 21), 71.4% exhibited LOM of IC2 as expected (Fig. 3b). The second most frequently hypomethylated locus was *GNAS* (63.6%). Other clinically relevant but least affected by LOM were *PEG3* and *SNRPN*. Seven individuals with MLID and BWS features exhibited LOM of both IC1 and IC2 (of note, the IC1 LOM is characteristic for SRS, IC2 LOM for BWS) (Fig. 4). Additionally, they all showed LOM at *MEST*, *PLAGL1* and *GNAS*. Other imprinted loci were affected less frequently.

As expected, for the 14 individuals referred with clinical suspicion of SRSp, LOM of IC1 was detected in the majority. The second most frequently hypomethylated locus was IC2. The loci least affected by LOM were *PLAGL1* and *SNRPN* (Fig. 3b). Six of the 14 MLID individuals with SRS features showed LOM at both IC1 and IC2 (Fig. 4). In these children, seven additional loci were hypomethylated and *MEST* (66.6%) and *GNAS* (50%) were the two mostly affected loci. For *ZFP57-*associated TNDM, there were two imprinted loci in addition to *PLAGL1* that commonly showed LOM (see above) (Fig. 3b).

## Discussion

In this study, we overview the available molecular and clinical data of 55 families (50 previously published and 5 new) with MLID associated with variants in *trans*-acting factors. In addition to 16 families with homozygous or compound heterozygous variants in *ZFP57* or a single homozygous variant in *ZNF445*, we identified 20 families with biallelic variants in maternal effect genes: *NLRP2*, *NLRP5*, *NLRP7*, *PADI6* and *KHDC3L*. In addition, there were 19 families where only one monoallelic variant had been identified in *NLRP2*, *NLRP5*, *NLRP7* or *PADI6.* The significance of a single heterozygous variant is currently unclear and requires further investigation. It is possible that, in these families, there is a second pathogenic variant that could not be detected by the molecular testing strategy used or the finding is coincidental. The possibility of multifactorial (e.g. monoallelic variants increasing susceptibility to environmental factors) or oligogenic inheritance cannot be excluded either. Thus, identification of a single monoallelic variant in a maternal effect gene should be considered cautiously and depending on the clinical suspicion such a finding might prompt more extensive genetic testing to search for a second *in trans* variant. Furthermore, modifying genetic variants in other genes, affecting the interactions between the members of the SCMC should be considered as these may add to the broad phenotypic spectrum observed in the patients.

The majority of the families with a genetic cause for MLID were linked to maternal effect genes encoding components of the SCMC (*NLRP2*, 7 families; *NLRP5*, 10 families; *NLRP7*, 5 families; and *PADI6*, 12 families), followed by *ZFP57* (16 families). *ZFP57*-linked families differed from those with maternal effect gene variants not only in the inheritance pattern but also in the clinical phenotype. *ZFP57* variants identified in the affected individuals were strongly associated with TNDM, whereas the offspring of the mothers with maternal effect gene variants most commonly presented with BWSp and SRS but also with non-specific features. Furthermore, due to the central role of SCMC in oocyte maturation and early embryonic development [22], disruption of one of the SCMC components can also predispose to pregnancy complications and developmental failure resulting in pregnancy loss and molar pregnancies (as observed for the affected families reported here).

The first evidence for contribution of SCMC gene variants to the aetiology of disturbed imprinting came in 2009 from a consanguineous family with a homozygous *NLRP2* variant and two children with BWSp and MLID [23]. However, it took several years before pathogenic variants in *NLRP5* and *NLRP7* were shown to cause MLID in congenital imprinting disorders [20, 24]. At that time, contribution of pathogenic *NLRP7* variants to the aetiology of recurrent BiHDM was already well established [25, 26], and together with *KHDC3L* these alterations in the mother were shown as the major causes for HDM. Recently, PADI6, another protein co-localized with the SCMC, has been associated with MLID. Thus, there is an emerging picture of consequences from biallelic maternal effect gene variants ranging from pregnancy loss, molar pregnancies to imprinting disorder phenotypes [12]) (Table 3).

The comparison of the maternal effect variants in MLID families with those identified in BiHDM cohorts confirmed this phenotypic transition as four of the seven variants in *NLRP7* had previously been identified in the latter group: p.(Met192Leu), p.(Phe250Cys), p.(Ala719Val), p.(Arg721Trp), p.(Ile858Thr) [27, 28] (https://infevers.umai-montpellier.fr/web/). It should be noted that p.(Met192Leu) variant has a relatively high frequency in gnomAD, and it should be regarded as a variant of unknown significance (VUS).

The relative frequency of the different phenotypes is likely to be influenced by both the functional effect of the gene and the gene variant. *NLRP7* and *KHDC3L* appear to be more commonly associated with BiHDM whereas *NLRP5*-linked MLID is more often observed in families with imprinting disorders. Hypomorphic *NLRP7* variants are more likely to be associated with viable pregnancies and complete loss of function variants are linked to more severe phenotypes [29]. As discussed above, the reports on mothers with a heterozygous maternal effect gene variant might reflect the possibility that they carry a second variant in the non-coding regions of the gene (e.g. introns, promotors or other regulatory regions) which are not detected by the current exon-focused sequencing approaches. However, there is currently no obvious difference of variant types between MLID families and families with recurrent reproductive failure.

In addition to the potential for a ‘missing *in trans*-variant’ in the mothers with a heterozygous maternal effect gene variant, there are multiple other factors which likely lead to under-diagnosis of variants in these genes and make the interpretation of variant pathogenicity challenging:

(a) Due to the non-traditional presentation of the disorder where the carrier mother will be clinically normal (except for their reproductive history), the possibility of a disorder associated with a maternal effect gene is overlooked.

(b) In many centres, MLID testing is not performed routinely and this situation might therefore escape detection. MLID testing is often initiated only when congenital imprinting disorder is recurrent in a family, in case of an atypical phenotype, or in case of simultaneous detection of LOM at IC1 and IC2 in the same affected individual [30].

(c) Several of the criteria defined by the American College of Medical Genetics and Genomics and the Association for Molecular Pathology (ACMG-AMP) [31], which are commonly used for the classification of a genomic variant, are not readily applicable for the classification of maternal effect variants as the carriers of the variants are asymptomatic and the variant-associated phenotype is presented in their offspring. Even biallelic pathogenic variants may occur without an obvious phenotype in men and females who did not had a pregnancy. Accordingly, the CADD Phred score provides only limited information due to the possibility that even pathogenic maternal effect variants might occur in control populations as it is the case with many autosomal recessive disorders.

A direct functional assay for assessing the pathogenicity of maternal effect gene variants would greatly aid variant interpretation, as no clinically applicable assays have yet been reported. The presence or absence of MLID in affected children/pregnancies would support pathogenicity. However, in addition to the fact that MLID testing is not part of the routine diagnostic flow in many centres other crucial determinants are still missing even if the testing is carried out. For example, there are not standardized criteria for which loci should be tested and what levels of LOM/GOM should be considered significant. We note that for variants in *ZFP57* there are epigenotype and phenotype correlations such that individuals with biallelic pathogenic variants demonstrate LOM at *PLAGL1*, *GRB10* and *PEG3*, and nearly all individuals show TNDM and associated features [32, 33]. ZFP57 contributes to a multiprotein complex that protects ICs from demethylation in the zygote, but the MLID pattern observed in TNDM/MLID individuals shows that it indeed has an impact on specific loci. This contrasts to the molecular and clinical findings in MLID carriers of maternal effect gene variants, for which specific gene episignatures are not obvious (Fig. 2a, b). We propose that detailed epigenotyping of MLID associated with maternal effect gene variants should be undertaken to determine whether specific episignatures can be defined [34]. Intriguingly, in families with *PADI6* variants more imprinted loci were hypomethylated than in families carrying variants in other maternal effect genes (Fig. 2). Though the number of the individuals is small, this observation is in line with the assumption that PADI6 plays a role in development in an earlier stage. An early embryonic arrest at the 2–4-cell stage has been demonstrated after in vitro fertilization of human oocytes carrying biallelic loss-of-function variants of *PADI6* [35]. *PADI6* variants can therefore be expected to cause a more severe epigenotype than the variants in factors which function later in embryogenesis. However, the smaller number of epimutations in the offspring of a mother with an *NLRP* variant might also be explained by the high homology between the *NLRP* genes which might allow a functional compensation.

Based on the molecular observations of the MLID families, different roles have been suggested for NLRP proteins in setting the imprinting marks. For NLRP7, an oocyte-specific function had been suggested in 2015, as only the maternally methylated loci seemed to be affected (for review: [36]). However, the identification of further MLID families showed that paternally methylated loci were also affected (Additional file 1: Table S1), suggesting that NLRP7 probably shared functional properties with NLPR2 and NLRP5 in the postzygotic maintenance of genomic imprinting. In conclusion, maternal effect variants can alter methylation of both maternally and paternally imprinted genes (for review: [37]).

In mothers with biallelic maternal effect gene variants, the recurrence risk of a child with MLID may be close to 100% (e.g. recurrent BiHDM with biallelic *NLRP7* variants) and ovum donation may be the only path to a normal pregnancy [29]. In families ascertained through a child with MLID, the recurrence risk for further pregnancies can be more variable and the phenotype is therefore difficult to predict (e.g. F7, F8, F25, Additional file 1: Table S1). Prenatal diagnosis for MLID by CVS (chorion villi sampling) or amniocentesis might be difficult to interpret, as in some families MLID can be detected in individuals with a normal phenotype and the finding of MLID with LOM at IC1 or IC2 might be associated with BWSp or SRSp [24]. Overall, BWSp individuals with MLID show a larger number of altered imprinted loci than those with SRSp (Fig. 3b). The majority of BWSp individuals with MLID have LOM of *GNAS*, *PLAGL1*, *GRB10* and *MEST*. In contrast, in SRSp individuals with MLID, IC2 is the most frequently affected additional locus. At least in BWSp, these patterns reflect the clinical overlap between the imprinting disorders associated with these loci: isolated *PLAGL1* LOM is associated with TNDM, and the TNDM MLID individuals caused by *ZFP57* variants show a phenotype with features similar to BWS (Additional file 1: Table S1). This overlap is confirmed by the family presented in this overview (F42), which was ascertained for molecular testing for BWS, but molecularly turned out to be a TNDM/*ZFP57* family. Furthermore, for the imprinted gene network a close functional link between PLAGL1 and IC2 has been demonstrated [10]. Currently, it is unclear why carriers with LOM of IC1 and IC2, can exhibit clinically opposite phenotypes. First data indicate that a more severe MLID signature seems to be associated with a BWSp phenotype to which LOM of *PLAGL1* and *GNAS* is linked to (Fig. 4).

The clinical descriptions of reported MLID cases depend on the age of the individual and the detail provided. For example, we do not have enough evidence to know whether the long-term development and prognosis of individuals with MLID differ significantly from individuals with isolated epigenetic errors. However, these assumptions are based on a small dataset, and further studies including larger cohorts are necessary to confirm these associations. Additionally, nearly all MLID data are based on genomic DNA from peripheral lymphocytes, but as a recent study on different tissues from SRSp individuals with epimutations has demonstrated, MLID individuals show a broad range of mosaic distribution of aberrant methylation patterns among different tissues [6].

Whereas the majority of MLID carriers show specific phenotypes associated with LOM of the disease-specific DMRs (primary epimutations, Table 1), the epigenetic pattern of other affected DMRs is more or less arbitrary, with a slight trend as described before: *PLAGL1*, *GRB10* and *GNAS* LOM rather appear to predispose to a BWS phenotype, though they can be altered in other imprinting disorders as well. For *MEST*, this type of apparent correlation is not obvious, and it is commonly and non-specifically hypomethylated (Fig. 2). In contrast, *SNRPN* is rarely affected in MLID. Considering the overall frequencies of LOMs for all loci, IC2 and *MEST* appear to be the most frequently affected, followed by *GRB10*, *PLAGL1* and *GNAS*. It can therefore be proposed that these two loci are the most vulnerable DMRs for disturbed imprinting maintenance. The reason for the vulnerability of specific loci is unclear, but the specific epimutation pattern in *ZFP57* variant carriers shows that different mechanisms for the establishment and maintenance of imprinting markers have to be considered. With the recent report on a homozygous variant in *ZNF445* in an MLID individual with a Temple syndrome phenotype, another promising candidate involved in imprinting resetting in the embryonic development has been suggested [19].

Due to the extensive genetic heterogeneity and the small number of MLID families reported so far, the conclusions drawn here should be regarded with caution. As long as a standardized methodology for MLID detection is missing, the comparison of data from different studies is limited. It is likely that genotype-epigenotype correlations might become obvious if more extensive methylation profiling is undertaken. Additionally, it should be noted that the focus on DNA from peripheral lymphocytes in routine diagnostics provides only a very restricted insight in MLID and its molecular spectrum. These limitations further complicate the compilation of MLID data, and in the future a consensus on MLID testing is needed. Finally, the studies to identify genetic trans-acting factors in MLID summarized here are based on different genetic approaches, ranging from Sanger sequencing to next-generation sequencing-based assays such as gene panels or clinical exomes. Accordingly, the functional impact of yet unidentified variants other than those published in the literature cannot be excluded.

## Outlook

The identification of genomic maternal effect and variants mutations causing MLID allows insights in the mechanisms behind the imprinting cycle of life, and the spatial and temporal function of the different factors during oocyte maturation and early development. Both basic research and identification of MLID families will help to understand the link between the different reproductive issues such as recurrent miscarriages and preeclampsia in maternal effect variant carriers/families and aneuploidy and MLID in the offspring. Though many questions remain to be answered, the current knowledge can already be used translationally for reproductive and genetic counselling in specific situations [12]. However, the basis for both research and counselling is a comprehensive catalogue of all molecular, clinical and reproductive data.

## Methods

A comprehensive literature search was conducted, using different keywords and combinations to identify families with genomic variants in genes associated with MLID and reported until August 2021. The keywords were: MLID, BWS, SRS, TNDM, *NLRP2*, *NLRP5*, *NLRP7*, *PADI6*, *ZFP57*, imprinting disturbance, maternal effect variant, maternal effect mutation and *NLRP* gene mutation. Families presenting only with molar pregnancies and/or pregnancy loss were excluded. Thereby 61 papers and an ESHG meeting abstract could be recorded. These papers were then evaluated for MLID cases associated with genomic variants in maternal effect genes and in *ZFP57*, and families from 21 papers and an ESHG 2021 abstract contribution (F48, F49) could be compiled (Additional file 1: Table S1). Additionally, four yet unpublished cases from the authors group could be included (F42, F53, F54, F55).

All information available about the families, the disease-associated variants, clinical findings, reproductive history and imprinting patterns were evaluated. The names of all identified 69 variants were checked by Mutalyzer (2.0.34), and some variants have been renamed according to HGVS. The total allele frequency and number of homozygotes were obtained from gnomAD v.2.1.1, for single variants allele frequencies were gathered from gnomAD v.3.1.1 (marked by ^a^ in Table 2). In silico pathogenicity prediction was carried out using Combined Annotation-Dependent Depletion (CADD, v1.6, https://cadd.gs.washington.edu/snv) (Table 2).

Due to the heterogeneous documentation of clinical data in the different studies, the clinical diagnosis was not traceable for all cases, therefore the terms BWS spectrum (BWSp) and SRS spectrum (SRSp) were used.

The comparison of the methylation patterns reported by the different studies is hindered by the lack of a commonly tested standard set of imprinted loci. We therefore decided to focus on the imprinted loci of clinical relevance, which were addressed in nearly all the reviewed studies. However, it should be noted that the nomenclature of imprinted loci is not used consistently in the literature. Thus, it is possible that even though the same imprinted locus was targeted, different CpGs were examined, as methylation-specific (MS) tests were not consistent and heterogeneous (e.g., pyrosequencing, multiplex ligation-dependent probe amplification (MLPA), PCR, array or bisulphite sequencing). For the SCMC encoding genes, genotypes of the mothers which have been ascertained as maternal effect variants were regarded as the cause for the MLID in the offspring, whereas for *ZFP57* and *ZNF445* only the affected individuals have been listed because they are the homozygous or compound heterozygous carriers of the gene variants. The presence of epimutations at two or more clinically relevant imprinting DMRs was considered to be diagnostic of MLID.

## Supplementary Information

**Additional file 1.** Overview on all cases, MLID patterns and list of references reporting on MLID associated maternal or fetal variants.

## Data Availability

The datasets generated and/or analysed during the current study are not publicly available due to privacy restrictions but are available from the corresponding author on reasonable request.

## Abbreviations

BiHDM: Biparental hydatidiform moles
BWS: Beckwith–Wiedemann syndrome
BWSp: BWS spectrum
CADD: Combined Annotation-Dependent Depletion (used as scaled Phred-line)
CNV: Copy number variant
DMR: Differentially methylated region
GNAS*: *GNA**S **A/B*:TSS-DMR; in some papers *GNAS-AS1*:TSS-DMR has been analysed (20q13)
GOM: Gain of methylation
GRB10*: *GRB10*:Alt-TSS-DMR (7p13)
HDM: Hydatidiform mole
IC: Imprinting center
IC1*: *H19/IGF2*:IG-DMR (11p15.5)
IC2*: *KCNQ1OT1*:TSS-DMR (11p15.5)
KOS14: Kagami–Ogata syndrome
MEG3*: *MEG3*:TSS-DMR (14q32)
MEST*: *MEST*:Alt-TSS-DMR (7q32)
MLID: Multilocus imprinting disturbance
LOM: Loss of methylation
PEG3*: *PEG3*:TSS-DMR (19q13.43)
PLAGL1*: *PLAGL1*:Alt-TSS-DMR (6q24)
SCMC: Subcortical maternal complex
SNRPN*: *SNRPN*:Alt-TSS-DMR (15q11)
SNV: Single nucleotide variant
SRS: Silver–Russell syndrome
SRSp: Silver–Russell spectrum
TNDM: Transient neonatal diabetes mellitus
TS14: Temple syndrome
UPD: Uniparental disomy
VUS: Variant of unknown significance

## Declarations

### Weblinks

- ClinVar: https://www.ncbi.nlm.nih.gov/clinvar/
- gnomAD: https://gnomad.broadinstitute.org/
- HGVS: https://varnomen.hgvs.org/
- Infevers: https://infevers.umai-montpellier.fr/web/
- LOVD: https://www.lovd.nl/
- Mutalyzer: https://mutalyzer.nl/
- MutationTaster: http://www.mutationtaster.org/
- SIFT: https://sift.bii.a-star.edu.sg/
